# Seroprevalence of Tick-Borne Encephalitis (TBE) Virus Antibodies in Wild Rodents from Two Natural TBE Foci in Bavaria, Germany

**DOI:** 10.3390/pathogens12020185

**Published:** 2023-01-25

**Authors:** Philipp Johannes Brandenburg, Anna Obiegala, Hannah Maureen Schmuck, Gerhard Dobler, Lidia Chitimia-Dobler, Martin Pfeffer

**Affiliations:** 1 Institute of Animal Hygiene and Veterinary Public Health, Faculty of Veterinary Medicine, University of Leipzig, An den Tierkliniken 1, 04103 Leipzig, Germany; 2 National Consulting Laboratory for TBE, Bundeswehr Institute of Microbiology, Neuherbergstrasse 11, 80937 Munich, Germany

**Keywords:** *Clethrionomys glareolus*, *Apodemus flavicollis*, epidemiology, tick-borne encephalitis, seroprevalence

## Abstract

Tick-borne encephalitis (TBE) is Eurasia’s most important tick-borne viral disease. Rodents play an important role as natural hosts. Longitudinal studies on the dynamics of the seroprevalence rates in wild rodents in natural foci over the year are rare, and the dynamics of the transmission cycle still need to be understood. To better understand the infection dynamics, rodents were captured in a capture-mark-release-recapture-study in two natural foci in Bavaria, Germany, monthly from March 2019 to October 2022. Overall, 651 blood and thoracic lavage samples from 478 different wild rodents (*Clethrionomys glareolus* and *Apodemus flavicollis*) were analyzed for antibodies against tick-borne encephalitis virus (TBEV) by indirect immunofluorescence assay (IIFA) and confirmed using a serum neutralization test (SNT). Furthermore, a generalized linear mixed model (GLMM) analysis was performed to investigate ecological and individual factors for the probability of infection in rodents. *Clethrionomys glareolus* (19.4%) had a higher seroprevalence than *A. flavicollis* (10.5%). Within *Cl. glareolus,* more males (40.4%) than females (15.6%) were affected, and more adults (25.4%) than juveniles (9.8%). The probability of infection of rodents rather depends on factors such as species, sex, and age than on the study site of a natural focus, year, and season. The high incidence rates of rodents, particularly male adult bank voles, highlight their critical role in the transmission cycle of TBEV in a natural focus and demonstrate that serologically positive rodents can be reliably detected in a natural focus regardless of season or year. In addition, these data contribute to a better understanding of the TBEV cycle and thus could improve preventive strategies for human infections.

## 1. Introduction

Tick-borne encephalitis virus (TBEV), belonging to the genus *Flavivirus* within the family *Flaviviridae*, is considered to be the most relevant tick-borne pathogen in Eurasia causing tick-borne encephalitis (TBE) [1,2,3]. Since 2012, TBE has been a notifiable disease in the European Union (EU) [4], resulting in over 15,000 registered human cases with an increasing incidence in the EU and the European Economic Area (EEA) between 2016 and 2020, with the highest number of confirmed cases reported in Czechia, Lithuania, and Germany [5]. In the year 2020, over 700 human cases were seen in Germany. Since then, the Robert Koch Institute (RKI) has expanded the number of risk areas to 175 in 2022. Risk areas are defined by the number of TBE cases reported in at least one of the 16 five-year periods from 2002–2021 in a district or district region (consisting of the district affected plus all neighboring districts) in comparison to the number of cases expected at an incidence of one disease per 100,000 inhabitants [6]. TBEV is divided into three genetic subtypes: the European (TBEV-Eur), the Far-Eastern (TBEV-FE), and the Siberian subtype (TBEV-Sib), with at least four other proposed subtypes: two Baikalian (TBEV-Bkl-1, TBEV-Bkl-2), the Himalayan (TBEV-Him) and the Obskaya subtype (TBEV-Ob) [7]. Most human infections with TBEV are asymptomatic. However, TBE can also manifest as fevers, acute progressive encephalitis, and debilitating neurological sequelae, and in less than 2% of cases, to death. TBEV thus has a significant impact on human health in endemic regions, including Central and Eastern Europe, Siberia, far-eastern Russia, northern China, and Japan [8,9,10].

All subtypes of TBEV are maintained in complex natural endemic transmission cycles, so-called microfoci with an average size of about 0.5–1 ha [11], involving ticks as natural vectors. In Central Europe, *Ixodes ricinus* ticks are the main vectors transmitting the European subtype to naïve hosts through blood meals after becoming infected with TBEV when feeding on a viremic host or co-feeding with an infected tick in close proximity to a non-viremic host [2,12,13]. In addition, they serve as a virus reservoir through transstadial and transovarial transmission within tick populations [14]. The main natural mammalian hosts of TBEV are rodents, in particular, the bank vole (*Clethrionomys glareolus*) and the yellow-necked mouse (*Apodemus flavicollis*) [3,12]. In endemic areas, recent studies propose a long-lasting or persistent infection in rodents [15,16] and the possible transmission of TBEV to their offspring via maternal milk, as described in a human case study [17]. In experimentally TBEV-infected voles, the virus persisted for more than three months in multiple organs and for 50 days in the blood, analyzed with real-time polymerase chain reaction (RT-qPCR) [18]. Humans become infected with TBEV via tick bite, through the consumption of infected unpasteurized milk or such milk products, or, in rare cases, through organ transplants [3,19].

Recent studies have highlighted the key role of voles as reservoir hosts for TBEV in natural foci. In several studies from Hungary, Slovenia, and the Czech Republic, the prevalence of TBEV in small mammals was monitored using different methods such as PCR, indirect immunofluorescent assay (IIFA), or serum neutralization test (SNT) [20,21,22]. In general, TBEV RNA can be detected by RT-qPCR during the viremic stage in several organs of the bank vole with the highest rates in whole blood, brain, and spine samples [23]. A comparative study of bank voles showed that thoracic lavage samples, in principle, allow the detection of neutralizing antibodies but showed a reduced sensitivity in comparison to serum samples. Therefore, the examination of thoracic lavage samples is also suitable if no serum sample is available [24]. In addition, the important significance of rodents, especially the bank vole, as part of the transmission cycle of TBEV was confirmed in the past decades. However, there is still a gap in our knowledge about the dynamics of the TBEV infection in a rodent population of a distinct TBE microfocus, i.e., the changing TBEV seroprevalence in rodents over a continuous period, the persistence of antibodies in rodents in nature, and demographic factors such as trapping location, year, season, species, age, and sex and their influence on the infection probability of the rodents in natural TBEV foci. Better knowledge of the transmission dynamics of the TBEV in foci might also help to predict periods with high TBEV prevalence in nature and, therefore, higher risk periods for infections in humans. Studies in the past have only partially addressed these questions. Field studies from TBEV natural foci in Hungary, Poland, and France have come to inconclusive results. They describe factors such as year, location, species, and age as having a possible influence on the TBEV prevalence in rodents, whereas season and sex of rodents had no influence [22,25,26]. However, these factors mentioned above have not yet been investigated together yet.

In the presented four-year study from 2019–2022, the wild rodent population from two well-described TBEV natural foci located in Bavaria, Germany, were serologically monitored for TBEV antibodies using a capture-recapture method to obtain data on the seasonal dynamics and to gain greater insight into the local ecology of TBEV transmission.

## 2. Results

### 2.1. Small Mammal Trapping

**Figure 1:**
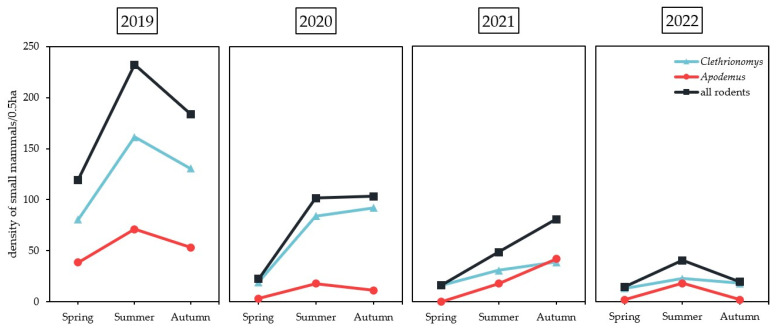
Calculation of the density in Haselmuehl and Heselbach for two species, the bank vole (*Clethrionomys glareolus*) and yellow-necked mouse (*Apodemus flavicollis*), per season and year. Density represents the minimum number alive (MNA) per season of captured rodents per 0.5 hectares. Spring (March–May), summer (June–August), autumn (September–October).

Overall, 706 captures of small rodents were documented (Table 1), corresponding to 500 different individuals (predominantly *Cl. glareolus*, n = 349, 69.8%, followed by *A. flavicollis*, n = 151, 30.2%). Furthermore, 100 individuals were re-captured 128 times within the two trapping nights of one trapping session, indicating no big harm for the animals to be trapped, chipped, and released again. Over the four years, the annual number of individually trapped bank voles and yellow-necked mice peaked in 2019 (*Cl. glareolus*, n = 189; *A. flavicollis*, n = 87), dropped in 2020 (*Cl. glareolus*, n = 99; *A. flavicollis*, n = 18), and decreased further in the two following years 2021 (*Cl. glareolus*, n = 47; *A. flavicollis*, n = 35), and 2022 (*Cl. glareolus*, n = 31; *A. flavicollis*, n = 13) (Figure 1). In the year 2019 (445 individuals/0.5 ha), the density of small mammals peaked during summer (June–August). In 2020 (189 individuals/0.5 ha) and 2021 (132 individuals/0.5 ha), the highest density was recorded in autumn (September and October), whereas in 2022 (71 individuals/0.5 ha), the density peaked again in summer. In every year and season, the density of *Cl. glareolus* was higher in comparison to *A. flavicollis*, except for autumn 2021 (Figure 1).

In total, 150 (30.0%) rodents were re-captured at two trapping sessions, and from these, 56 (11.2%) were even re-captured at a third trapping session. Overall, 19 (3.8%) rodents (17 *Cl. glareolus* and two *A. flavicollis*) were captured during two different years, with the longest period of 11 months between the trapping sessions (first capture July 2019 respectively June 2020, the last capture June 2020 and May 2021), and the shortest period of 5 months (first capture October 2019, last capture March 2020). The average period of rodents captured in two different years was 7.4 months. The proportion of rodents captured during two different years was 4.3% in 2020 (n = 12), and for the two following years, 3.8% in 2021 (n = 4) and 2022 (n = 3).

### 2.2. TBEV Seroprevalence in Small Mammals

We examined 420 serum samples (first capture: n = 280; second capture: n = 95; third capture: n = 45) as well as 50 thoracic lavage samples (first capture: n = 38; second capture: n = 11; third capture: n = 1) from 338 *Cl. glareolus* and 167 serum samples (first capture: n = 128; second capture: n = 29; third capture: n = 10) along with 14 thoracic lavage samples (first capture: n = 11; second capture: n = 3) from 140 *A. flavicollis*. Each serum and thoracic lavage sample belonged to different rodents, captured at one to three trapping sessions. Overall, 16.9% (95% CI: 13.8–20.6%, n = 81) of individuals, as well as 16.9% (95% CI: 14.2–20.0%, n = 110) of all tested samples were seropositive for TBEV antibodies. The proportion of seropositive individuals for *A. flavicollis* was 11.4% (95% CI: 7.1–17.9%, n = 16), and for *Cl. glareolus,* 19.2% (95% CI: 15.4–23.8%, n = 65). The proportion of entire seropositive samples was significantly higher in *Cl. glareolus* (19.4%, 95% CI: 16.0–23.2%, n = 91) compared to *A. flavicollis* (10.5%, 95% CI: 6.8–15.9%, n = 19) (Table 1) looking at the GLMM (*p*-value = 0.0005). The GLMM confirmed this effect on the individual infection probability by small mammal species for the location Haselmuehl (*p*-value = 0.0011) but not for the location Heselbach (*p*-value = 0.4047) (Table 2). The proportion of seropositivity did not differ significantly between years when looking at post hoc analysis (*p*-value = 0.361–0.986), varying from 11.8% (95% CI: 6.9–19.3%, n = 13) in 2021 to 19.2% (95% CI: 13.7–26.3%, n = 29) in 2020. Seroprevalence levels did differ significantly between summer (20.2%, 95% CI: 15.9–254%, n = 56) and autumn (10.8%, 95% CI: 7.6–15.2%, n = 29) (*p*-value = 0.0185) but looking at the post hoc analysis it was not significant for *A. flavicollis* (*p*-value = 0.278–0.879) and *Cl. glareolus* (*p*-value = 0.198–0.996). Consequently, spring, summer, and autumn seasons had no significant effect on the infection probability in small mammals (Table 2 and Table 3). TBEV antibodies were detected from spring to autumn in each year examined, except for the spring and autumn of 2022 (Table 1). Further, the GLMM showed that there were no differences in prevalence between small mammals from Haselmuehl (15.7%, 95% CI: 11.8–20.5%, n = 43) and Heselbach (17.8%, 95% CI: 14.2–22.0%, n = 67) (*p*-value = 0.2211) (Table 2 and Table 3). Seropositivity of sexes did differ significantly in the GLMM between female *A. flavicollis* (18.5%, 95% CI: 10.7–29.7%, n = 12) and male (7.1%, 95% CI: 3.0–14.8%, n = 6) (*p*-value = 0.0376). In *Cl. glareolus,* a significantly higher proportion of seropositivity was observed in males (40.4%, 95% CI: 31.8–49.5%, n = 46) than in females (15.6%, 95% CI: 10.9–21.8%, n = 27) (*p*-value = 2.43 × 10^−6^) (Table 2 and Table 3). Adult *A. flavicollis* (12.0%, 95% CI: 7.6–18.3%, n = 18) showed a significantly higher seroprevalence than juvenile (3.2%, 95% CI: 0.0–17.6%, n = 1) (*p*-value = 0.0374). In the GLMM for *Cl. glareolus*, the factors of age and sex correlate with each other, which is why both must be considered individually. As with *A. flavicollis*, adult *Cl. glareolus* (25.4%, 95% CI: 20.7–30.8%, n = 73) showed a significantly higher seroprevalence than juvenile (9.8%, 95% CI: 6.2–15.1%, n = 18) (*p*-value = 0.0004) (Table 2 and Table 3).

In total, 81 individuals were seropositive in at least one trapping session. Among them, 13 seroconversions were observed (four re-captured once positive), 12 seropositive rodents were re-captured once positive, and seven were re-captured twice positive. Among the seropositive recaptures, TBEV antibodies were detected in a maximal period of 189 days for *Cl. glareolus* and 69 days for *A. flavicollis*. Three seropositive rodents were tested seronegative at the time of recapture (36–107 days between captures) (Table 4).

### 2.3. Movement Profile of the Recaptured Small Mammals

**Figure 2:**
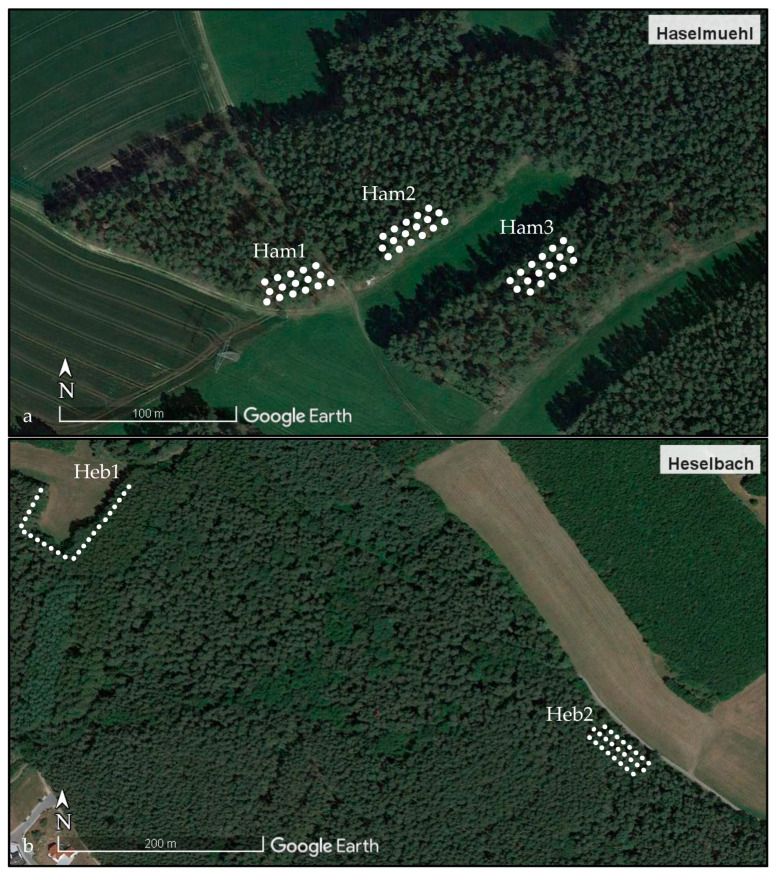
Overview of the sampling site in Haselmuehl with three sub-plots (Ham1–Ham3) (**a**) and Heselbach with two sub-plots (Heb1 and Heb2) (**b**); each white dot represents one live animal trap. The image was created by using Google Earth Pro, Map: Google Earth ©2022 Google, Image Landsat/Copernicus ©2022 GeoBasis-DE/BKG.

In the sub-plot Ham1 (size of 333 m^2^), 42 *Cl. glareolus* and six *A. flavicollis* were recaptured; in Ham2 (size of 366 m^2^), 28 *Cl. glareolus* and 13 *A. flavicollis* were recaptured; in Ham3 (size of 480 m^2^), 48 *Cl. glareolus* and four *A. flavicollis* were recaptured. In the sub-plot Heb1 (size of 330 m^2^), 26 *Cl. glareolus* and 23 *A. flavicollis* were recaptured, and in Heb2 (size of 1584 m^2^), 95 *Cl. glareolus* and 49 *A. flavicollis* were recaptured (Figure 2). In total, 141 recaptures were counted for the study site of Haselmuehl and 193 for the study site of Heselbach. Recapture has never occurred in two different sub-plots. Within the sub-plots, the range of movement was distributed over the entire sub-plot.

## 3. Discussion

This four-year study increases our knowledge of the seasonal and interannual dynamics of TBEV circulation in small mammals in two active natural TBE microfoci and provides additional information on demographic host factors influencing TBEV infection on an individual level. Our results confirm an active circulation of TBEV in rodents of at least two different species in two natural TBE foci. This study is the first to examine small mammals for the dynamics of TBEV antibodies over four years in Germany. The two TBE foci, Haselmuehl and Heselbach, were identified as TBE microfoci after residents near the foci developed clinical TBE symptoms. Since 2009 TBEV was continuously detected in questing ticks in these foci [27,28]. Both sites are located in one of the most severely TBE-affected areas in Germany, with the highest incidence rates in the current five-year period (2017–2021) with a total of 115 reported human cases in both districts combined [29].

*Apodemus flavicollis* and *Cl. glareolus* densities (71–445 individuals/year/0.5 ha) at those sites were investigated in the current study and were comparable to densities from a TBEV seroprevalence study in Hungary (78–165 individuals/year/0.5 ha) [30] and substantially higher than data from France (4–57 individuals/year/0.5 ha) [27]. The abundant food supply of fruits and fungi via seeds from seed-rich trees in an area is essential to maintain rodent population densities [31]. These environmental conditions seemed to be adequate in our study areas to maintain stable rodent populations over the study period. To successfully circulate TBEV in nature, the virus requires an area with dense rodent and tick populations and woodlands with dense understory cover, which are present at both study sites [27,32].

For the continuous detection of TBE infection in rodents, serological test systems are usually the methods of choice as virus detection by PCR or virus isolation usually needs organ material which causes the death of the animal. The SNT is considered the most specific serological test; however, it needs to work with live viruses under BSL-3 laboratory conditions [33]. Whereas methods such as the IIFA, which we mainly used in our study, were shown to reach a sensitivity of almost 80% and a specificity of nearly 99% compared to the neutralization test (NT) when tested with dog sera [34] such comparative studies in sera from wild rodents are missing. Possible cross-reactions with other flaviviruses, such as West Nile virus or Louping-ill virus, are negligible since rodents are not natural reservoirs in the enzootic cycles of these viruses and are not known to be prevalent in the study areas [35]. We took the opportunity to test if the IIFA may be a suitable alternative for the detection of antibodies against TBEV in small mammals. Therefore, all thoracic lavage samples from the years 2019–2022 that had tested positive using IIFA were verified by SNT and showed a sensitivity of 94.3% compared to SNT results. There is a possibility that specific TBEV antibodies may be detected by IIFA that do not show neutralizing activity and therefore are not detected by NT, which could explain the slightly lower detection rate [34].

In our study, TBEV antibodies were detected in 16.9% of the investigated samples. The seroprevalence in the current study is higher than that observed in studies in Northern and Central Europe that have also examined rodent samples serologically using IIFA. In two natural foci in Finland, a seroprevalence of 4.0% was found [15], while nationwide studies from Slovenia [21], Germany [18], and Switzerland [36] showed seroprevalences of 5.9%, 10.2%, and 3.6%, respectively. Other serological studies using different testing methods from natural foci in Western and Central Europe published seroprevalences of 5.1% in Hungary [30], 14.8% in Poland [25], 1.5% in the Czech Republic [37], 7.0% and 14.6%, respectively, in Slovakia [38,39], 4.2% in France [26], and 14.0% in Bavaria, Germany [40], while a study from Eastern Europe in Russia described a seroprevalence of 61.4% in small mammals in a study area with occurrences of the TBEV-FE, and the TBEV-Sib which may explain this high prevalence [16]. Concerning the seroprevalence rates, our results are to be classified for the bank vole (19.4%) in the highest prevalence range within Europe (2.2% to 20.5%) [15,18,21,22,25,26,36,37,38,39,40], with for example, 14.3% in Slovenia [21], 5.3% in Switzerland [36]. For the yellow-necked mouse (10.5%), the prevalence varies in the middle range (1.3% to 18.1%) [18,21,22,26,36,37,38,39,40], with, for example, 4.0% in Slovenia [21] and 18.1% in Slovakia [39].

In Haselmuehl, yellow-necked mice seem to play a minor contribution to TBEV circulation (1.6%). In Heselbach, seropositive, yellow-necked mice were found only in 2019 (22.6%) and 2020 (20.0%), but none at all in 2021, and only one individual in summer 2022 (7.7%), which is indicative of a subordinate role concerning the circulation of the virus within the natural foci studied for this rodent species. Similar background positivity for *A. flavicollis* was described in Hungary (3.7%) [22] and in Switzerland (1.3%) [36]. The fact that the TBEV prevalence is higher in bank voles has also been pointed out earlier [15,18,21,25]. A study from France described a significantly higher tick load on *A. flavicollis* compared to *Cl. glareolus* [26], which may develop resistance to feeding ticks after repeated infestations through the involvement of T-helper cells in the immune response. Furthermore, due to the larger body surface, *A. flavicollis* seem to be more susceptible to tick infestation [41]. This finding should lead to a higher prevalence of *A. flavicollis*. Yet, evidence of stronger viremia, higher antibody titers, and a longer half-life of TBEV antibodies in *Cl. glareolus* compared to *Apodemus* species disproved these assumptions, presumably due to immune response characteristics to TBEV infection [21,42,43,44]. Thus, the above-shown data indicate that *Cl. glareolus* is more significant compared to *A. flavicollis* for a systemic transmission of TBEV.

Another finding of our study was that within the species *Cl. glareolus, a* significantly larger number of males (40.4%) were seropositive compared to females (15.6%). One reason for this could be the larger territories of male bank voles and, thereby, the possible increased contact with a higher number of ticks in the vegetation [22,45]. Due to high testosterone levels, sexually active bank voles also exhibit reduced innate and acquired immunity, allowing more ticks to be collected and increasing the transmission probability of TBEV [46].

Host age also plays a vital role in our study, which significantly affects the seroprevalence of TBEV in rodents. This observation was also confirmed by other studies in Hungary and Poland [22,25]. During the period of investigation, the seroprevalence of the virus was higher among adult bank voles and yellow-necked mice (25.4%, respectively 12.0%) compared to juvenile individuals (9.8%, respectively 3.2%). Also, in Hungary and Poland, more adult bank voles (25.9%, respectively, 20.8%) were seropositive compared to juveniles (8.3%, respectively 8.7%) [22,25]. The likelihood and possibilities of parasitic infestation increase with host age [47]. In addition, seroprevalence increases with age because antibodies have been detected over a long period (189 days). Nevertheless, these results must be interpreted with caution since we used only weight as a parameter for age classification and had three individuals that were seronegative when recaptured but seropositive before. Another limitation of the study is that we also cannot clarify by the study design whether seropositivity is maintained by a single TBEV infection or by recurrent bites of infected ticks.

From 2019–2022, we did not detect significant annual or seasonal differences in TBEV seroprevalence in rodents, although the density of small mammals has decreased over the years. A higher seroprevalence could be expected in years with fewer rodents. Thus, an increased tick load per individual, which did not occur as described in France, where a total of 541 small mammals were sampled in 2012, 36 in 2013, and 87 in 2014, and the seroprevalence had dropped from 3.5% to 1.1%. In contrast, the tick infestation rate on rodents increased from 38.2% to 83.3% over the years [26,36]. It is known that antibodies against TBEV are detectable in the blood of rodents after 5 days post-infection [18,23]. In several studies, TBEV antibodies were detected up to 100–168 days after infection [18,48]. In our study, all but three rodents tested seropositive continuously in re-captures with the longest period of up to 189 days, indicating a very long circulation period of TBEV antibodies. The fact that two of the three rodents shown to have converted from seropositive to seronegative during recaptures were *A. flavicollis,* and the detection period of seropositivity was shorter than that of *Cl. glareolus* may also be a reason for the lower seroprevalence in *A. flavicollis*. The detection of seroconversions across all seasons studied, even between years, indicates that rodents are likely exposed to year-round infection pressure. Since *Clethrionomys* spp. have 3–4 litters during the reproductive season from April to the end of September and *Apodemus* spp. have 2–3 litters from March to the end of October, naïve animals are always present in the natural focus which can become infected, and thus serve as a transmission source of TBEV for juvenile tick stages during the viremic phase of up to 28 days post-infection [23,49]. Due to the short lifespan of rodents (maximum 11 months in our study) and the low annual recapture rates (3.8–4.3%), it can be inferred that a new naive population builds up each year in spring and is infected by ticks in which the virus has overwintered. Similar data were described in a seroprevalence study of recaptured rodents from Hungary, where the maximum lifespan was 1–1.5 years and the recapture rates from last year ranged from 1.3–7.3% [30].

The movement profiles of the recaptured animals were particularly interesting in the study site of Haselmuehl because the three sub-plots were close to each other (distance of 30–40 m). It turned out that among the 141 recaptures, not a single animal was caught in two different sub-plots. In the literature, home ranges of *Cl. glareolus* are described from 737–1753 m^2^ and for *A. flavicollis* 100–2300 m^2^ [50,51]. Thus, as in Haselmuehl, a footpath or a meadow represent natural barriers for the rodents, which will not be crossed under optimal living conditions. From this, we can infer that the virus cycle between juvenile ticks and rodents occurs in several small microfoci with an average size of about 0.5 to 1 ha forming a natural focus. From these microfoci, infected adult ticks passively migrate with the help of larger wild animals to other locations to form a new microfocus under adequate conditions, which would be, i.e., formed by the presence of vector and reservoir hosts, suitable climatic conditions for the vitality of *I. ricinius* and the presence of coniferous or mixed forests [11].

## 4. Materials and Methods

### 4.1. Study Area

**Figure 3:**
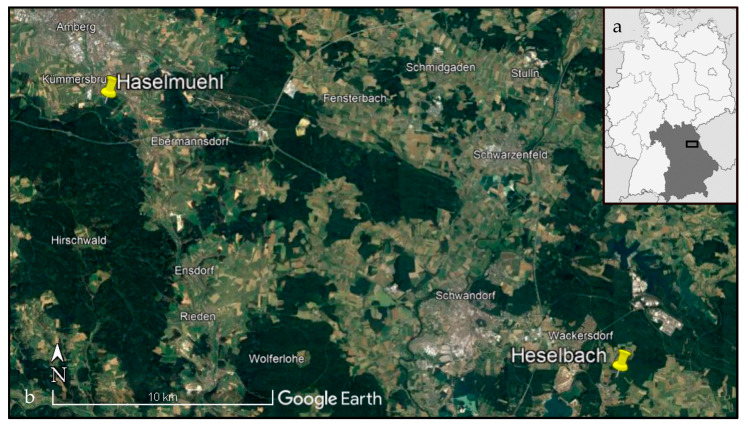
Overview of Bavaria (dark grey) in Germany (white) with the location of the study area marked by a square (**a**); trapping locations within the study area (**b**). The image was created by using Google Earth Pro, Map: Google Earth ©2022 Google, Image Landsat/Copernicus ©2022 GeoBasis-DE/BKG.

The study was conducted in two well-studied natural foci of TBE at Haselmuehl and Heselbach, where viral sequences were previously obtained from TBEV strains from questing ticks [28]. The first site, “Haselmuehl,” is a rural area in the administrative district of Amberg-Sulzbach, around 60 km east of Nuremberg, in the German federal state of Bavaria (Figure 3). It is located 430 m above sea level at a geographic longitude of 11°52′53.6″ E and a latitude of 49°24′31.0″ N. The sampling site is divided into three sub-plots (Ham1–3) separated through a footpath or a meadow, which have a size of 0.03 to 0.05 ha and are close to each other (Figure 2). The second site, “Heselbach,” is a rural area in the administrative district of Schwandorf, more than 26 km southeast of Haselmuehl, in the German federal state of Bavaria (Figure 3). It is located 440 m above sea level at geographic longitude 12°12′02.4″ E and latitude 49°17′51.2″ N. This sampling site is divided into two sub-plots (Heb1 and 2) with a distance of 500 m in between, which have a size of 0.02 and 0.15 ha (Figure 2). The sampling sites in Haselmuehl and Heselbach are characterized by mixed forests with primary pines (*Pinus sylvestris*), fern species, hazelnut, broom, and blackberry bushes. Both study sites belong to the two most affected TBE districts in Germany concerning the current five-year incidence (2017–2021), with an incidence of 55.34/100,000 inhabitants for the district of Amberg-Sulzbach, and an incidence of 40.96/100,000 inhabitants for the district of Schwandorf [6].

### 4.2. Small Mammal Trapping and Sampling

Small mammals were trapped for 8 months per year from March to October in the years 2019–2022, except for April and May 2020, when rodent trapping was not possible due to travel restrictions during the COVID-19 pandemic. The trapping grid consisted of 50 live animal traps (sized 7.62 × 8.89 × 22.86 cm, H. B. Sherman Inc., Tallahassee, FL, USA) per study area set in lines at 5–15 m intervals covering the sub-plots, if possible, over a rodent hole or near a tree trunk (Figure 2). Each trap is assigned a number, which is recorded on the trap log of the respective trap location. Live traps were set for two consecutive nights and baited with peanut flips and apple pieces. Wood wool was placed in the traps to provide nesting material and to prevent hypothermia (overall 30 trapping sessions, 60 trapping nights). The traps were checked in 12-h intervals in the morning and evening. The captured rodents were placed individually in a bucket and anesthetized with isoflurane at a 5% concentration and an oxygen flow of 1 L/min until the motorial movement of the rodent was no longer observed. Subsequently, inhalation anesthesia was maintained with isoflurane at a 2.5–3.5% concentration and an oxygen flow of 1 L/min to reduce stress during handling and sampling. At first capture, each rodent was individually marked with a transponder (Glass transponder EM4102, 2.12 × 12 mm, LUX-IDent s.r.o., Lanškroun, Czech Republic). Trapping location and date of capture, species, sex, reproductive status (testicles visible for males; vagina open, teats formed for females), body mass, and length were recorded, ectoparasites collected located on the rodent, and 100–200 µL of blood was taken through the retro-orbital sinus from rodents weighing over 14 g. By documenting the exact trapping location within the sub-plots of the study sites, we could create movement profiles of the re-captured animals to make a statement about the distances covered. The rodents were divided into two age groups (juvenile and adult) instead of three based on weight and time between the re-captures (age class 1 and 2, respectively 1 comprise juvenile *Cl. glareolus*, respectively *A. flavicollis*; age class 3 respectively age class 2 and 3 comprise adult *Cl. glareolus* respectively *A. flavicollis*) [52]. Accordingly, individuals weighing less than 19.5 g (less than 2.5 months old) for *Cl. glareolus* and 20 g for *A. flavicollis* (less than 3.5 months old) were considered juveniles. When the time between the re-captures was longer than 2.5 months for *Cl. glareolus*, respectively 3.5 months for *A. flavicollis*, the rodent was classified as an adult. The number of captures and the number of samples were not equal since some animals were not sampled due to weakened physical conditions or the body weight was below 14 g. After sampling, the rodents were placed back into the bucket. Anesthetic treatment was stopped, and rodents were observed. Animals that were visibly fully awake were released at the exact location of capture, and traps were re-baited. For rodents that were re-captured within a trapping session, only the location of capture was documented and afterward directly released. Once a rodent was captured in three different months, it was euthanized by exsanguination through the retro-orbital sinus, followed by neck fracture under anesthesia, as described above. Euthanized rodents and rodents that died in the traps were immediately stored on dry ice (−80 °C). Together with the blood samples, stored at +4 °C, they were transported to the laboratory for further processing. In the laboratory, the blood samples were centrifugated at 7000 rpm for 8 min, and serum samples were obtained from the supernatant. A thoracic lavage with 500 µL phosphate-buffered saline buffer (PBS) was taken from rodents that died in the trap. Serum and thoracic lavage samples were frozen at −80 °C until testing for TBEV antibodies.

### 4.3. Ethical Statement

The animal experiment with small mammals fulfilled the EU Directive 2010/63/EU and was approved by the District Government of Lower Franconia (RUF-55.2.2-2532-2-780-15). All efforts were made to minimize animal suffering. *Apodemus flavicollis* is a protected species in Germany according to Section 7, paragraph 2, Section 13, letter c) of the Federal Nature Conservation Act (BNatSchG) in connection with attachment 1 of the Federal Species Protection Regulations (BArtSchV). The exemption for this study was approved by the District Government of Upper Palatinate (ROP-SG55.1-8646.4-1-96-19). Only trained staff was handling the animals under EU directive 2010/63 Function A and following the recommendations of the Federation of European Laboratory Animal Science Associations (FELASA) and the Society of Laboratory Animals (GV-SOLAS). The rodent trapping took place with permission from the landowners.

### 4.4. Serological Analysis

#### 4.4.1. Indirect Immunofluorescence Assay (IIFA)

Serum and thoracic lavage samples were transported on dry ice to the Bundeswehr Institute of Microbiology (Munich, Germany) and were screened for the presence of TBEV antibodies using an IIFA (FSME-Viren (TBEV), Euroimmun AG, Luebeck, Germany). The testing was performed according to the manufacturer’s instructions with appropriate adaption for examining rodent samples instead of human samples, as it has already been performed with dog samples [34]. Therefore, the enclosed fluorescein-labeled anti-human conjugate was replaced with an anti-mouse conjugate (DAKO, Glostrup, Denmark) and used in a pre-defined 1:20 dilution. Serum samples were diluted 1:10, according to the dilution scheme to determine antibodies of the IgG class. Thoracic lavage samples were used without dilution, assuming a dilution of about 1:10 before the examination for TBEV-specific antibodies. The results were read independently by two trained staff members using a fluorescence microscope (Leica DM 5000B, Wetzlar, Germany) and classified as either “positive” (fine to coarse granular structures fluoresce in the cytoplasm, no fluorescence in the control field) or “negative” (no fluorescence in the cytoplasm visible, no fluorescence in the control field/uncharacteristic fluorescence in positive and control field), as recommended by the manufacturers.

#### 4.4.2. Serum Neutralization Test (SNT)

To avoid false positive samples, the IIFA-positive thoracic lavage samples of 2019–2022 were confirmed with the SNT, which was not used for serum samples due to the insufficient amount of serum obtained by retro-orbital puncture. The SNTs were performed according to standard procedures [53] using the validated protocol of the accredited diagnostic laboratory at the Bundeswehr Institute of Microbiology (Munich, Germany) [54]. In summary, TBEV (strain Neudoerfl) was cultured in A549 cells, and virus stocks (40–60 tissue culture infective dose (TCID)/50 µL) were prepared and stored at −80 °C until further use. SNTs were performed in a micro-format in 96-well cell culture plates (Greiner bio-one, Frickenhausen, Germany). After inactivation of thoracic lavage samples at 56 °C for 30 min, they were run in duplicate and diluted in Minimal Essential Medium (MEM, plus Non-Essential Amino Acids Solution plus Antibiotic-Antimycotic Solution; all Invitrogen, ThermoFisher Scientific, Darmstadt, Germany). Assuming a predilution of about 1:10, the dilutions ranged from 1:20–1:2560. One cell control and one virus re-titration were used as controls on each 96-well plate. A total of 40–60 TCID of virus stock was added to each well, and the respective thoracic lavage-virus solutions were incubated at 37 °C (5% CO_2_) for one hour. A549 cells (1 × 10^4^ cells/50 µL) were then added per well and incubated at 37 °C (5% CO_2_) for 5–7 days. The supernatant was then discarded, and the 96-well plates were fixed in 13% formalin/PBS, stained with crystal violet (0.1%), and the titers were determined visually. The antibody titer corresponding to the highest thoracic lavage dilution that showed complete inhibition of cytopathic effect (CPE) in both wells was reported. Samples were classified as either “SNT negative” (titer < 1:20) or “SNT positive” (titer ≥ 1:20), with the highest readable titer being ≥ 1:2560.

### 4.5. Statistical Analysis

#### 4.5.1. Definition

The trapping sessions from March to October 2019 to 2022 were divided into three seasons: spring grouped the captures of March to May (beginning of small mammal reproduction); summer corresponded to the captures of June to August (peak of small mammal reproduction); and autumn grouped the captures of September and October (reduction of small mammal abundance).

The calculation of the density is based on the minimal number alive (MNA), i.e., the number of individual rodents captured in a season plus the number of rodents captured in at least one previous and one following season to estimate the population size. The number of captures was divided by the size of the sampling sites in the unit ha and extrapolated to 0.5 ha for better illustration and comparison.

#### 4.5.2. Statistical Analyses of TBEV Seroprevalence in Small Mammals

Confidence intervals (95% CI) for the prevalence were determined by the Clopper and Pearson method with GraphPad Software (Graph Pad Software InCr., San Diego, CA, USA). To analyze TBEV prevalence in small mammals in relation to season, year, habitat, and small mammal species, we conducted a generalized linear mixed model (GLMM) with binomial error distribution using R-software (version 4.1.2. for Windows, Boston, MA, USA) and the lme4 package [55]. The infection status was used as a binary dependent variable (TBEV-seropositive = 1; TBEV-seronegative = 0). The GLMM was generated to estimate how (1) seasonality (independent binary variable: summer vs. spring); (2) small mammal species (independent categorical variable); (3) host age (independent binary variable: adult vs. juvenile), and (4) sex (independent binary variable: male vs. female), (5) year of capture (independent categorical variable), and (6) habitat (independent binary variable: Haselmuehl vs. Heselbach) affect individual infection status (dependent binary variable). For small mammals, the interaction term for the GLMM consisted of three variables with at least two levels each. Therefore, we computed marginal means using the emmeans package within R and a post hoc test for comparing the effects of all independent variables separately [56]. The significance threshold was set at *p* ≤ 0.05.

## 5. Conclusions

This study presents for the first time the seroprevalence rates of TBEV antibodies in wild rodents in the two natural foci of Haselmuehl and Heselbach in Germany over four years. TBEV antibodies were detected at an average prevalence rate of 16.9% in rodent sera and thoracic lavage, irrespective of seasonal or annual variation. This was regardless of the detection of seroconversions across seasons and between years. This confirms the presence of TBEV at suspected sites in reservoir hosts and the possibility of TBEV infection in rodents throughout the year. Antibodies were detected in a maximum period of 189 days for *Cl. glareolus* and 69 days for *A. flavicollis,* indicating a very long, maybe life-long period of circulating TBEV antibodies. Male adult bank voles were more often infected with TBEV in our study. Yellow-necked mice probably play a subordinate role as hosts in the TBEV cycle. Thus, the probability of infection of rodents depends rather on individual factors such as species, age, and sex than on abiotic and biotic external factors such as study site of a natural focus, year, and season. More studies of this type on other TBEV natural foci and reservoir hosts, as well as experimental studies with rodents and ticks, are necessary to better understand the time of infection and seroconversion for *Cl. glareolus* and *A. flavicollis* and the period of seropositivity as a contribution to a better understanding of the complex life cycle of TBEV.

## Figures and Tables

**Table 1:** Tick-borne encephalitis virus seropositivity rates for rodents by years, seasons, and locations. Serum and thoracic lavage samples are considered together.

		Haselmuehl								Heselbach							
		*Clethrionomys glareolus*				*Apodemus flavicollis*				*Clethrionomys glareolus*				*Apodemus flavicollis*			
Year	Season	NTR	Seropos. (Seropos./ Sampled Rodents	RF (%)		NTR	Seropos. (Seropos./ Sampled Rodents	RF (%)		NTR	Seropos. (Seropos./ Sampled Rodents	RF (%)		NTR	Seropos. (Seropos./ Sampled Rodents	RF (%)	
				Y	S			Y	S			Y	S			Y	S
2019	Spring	34	7/30	21.3	23.3	11	0/10	0	0	28	7/26	18.0	26.9	13	4/10	22.6	40.0
	Summer	72	14/59		23.7	12	0/11		0	49	5/32		15.6	38	8/33		24.2
	Autumn	52	9/52		17.3	17	0/17		0	42	6/42		14.3	21	2/19		10.5
2020	Spring	0	0/0	10.8	0	0	0/0	0	0	7	3/7	23.9	42.9	0	0/0	20.0	0
	Summer	19	2/19		10.5	5	0/5		0	49	14/44		31.8	11	3/11		27.3
	Autumn	19	2/18		11.1	3	0/2		0	41	5/41		12.2	4	0/4		0
2021	Spring	2	0/2	17.6	0	0	0/0	5.6	0	11	4/11	18.8	36.4	0	0/0	0	0
	Summer	2	1/2		50.0	1	0/1		0	22	3/22		13.6	11	0/11		0
	Autumn	13	2/13		15.4	17	1/17		5.9	15	2/15		13.3	16	0/16		0
2022	Spring	6	0/6	33.3	0	0	0/0	0	0	3	0/3	0	0	1	0/1	7.7	0
	Summer	9	5/9		55.6	1	0/1		0	6	0/6		0	11	1/11		9.1
	Autumn	0	0/0		0	0	0/0		0	11	0/11		0	1	0/1		0
Total		228	42/210	20.0		67	1/64	1.6		284	49/260	18.8		127	18/117	15.4	

Y = Year; S = Season; NTR = Number of trapped rodents; Seropos. = Seropositivity; seropos. = seropositive; RF = Relative frequency.

**Table 2:** Results of a generalized linear mixed model with binominal error distribution with effects of location, seasonality, small mammal species, sex, and age on infection probability in small mammal specimens in total, per location and species.

Factor	Estimate	Std. Error	z-Value	*p*-Value
Total				
Intercept	−2.9372	0.3989	−7.363	1.8 × 10^-13^ ***
*A. flavicollis* v. *Cl. glareolus*	1.0603	0.3037	3.491	0.000481 ***
Autumn v. spring	0.6671	0.3517	1.897	0.057849 .
Autumn v. summer	0.6463	0.2745	2.355	0.018545 *
Haselmuehl v. Heselbach	0.2883	0.2356	1.224	0.221094
2019 v. 2020	−0.1045	0.2798	−0.374	0.708743
2019 v. 2021	−0.5708	0.3552	−1.607	0.108080
2019 v. 2022	−0.7571	0.4762	−1.590	0.111845
Adult v. juvenile	−0.6716	0.3426	−1.960	0.049969 *
Female v. male	0.8102	0.2509	3.229	0.001240 **
Haselmuehl				
Intercept	−5.88806	1.16148	−5.069	3.99 × 10^−7^ ***
Autumn v. spring	0.04609	0.58515	0.079	0.93722
Autumn v. summer	0.54246	0.43445	1.249	0.21181
*A. flavicollis* v. *Cl. glareolus*	3.44187	1.05740	3.255	0.00113 **
2019 v. 2020	−0.65451	0.61951	−1.056	0.29074
2019 v. 2021	0.51385	0.66947	0.768	0.44276
2019 v. 2022	0.09561	0.68152	0.140	0.88843
Adult v. juvenile	0.41223	0.51703	0.797	0.42528
Female v. male	2.11164	0.47819	4.416	1.01 × 10^−5^ ***
Heselbach				
Intercept	−1.73161	0.49816	−3.476	0.000509 ***
Autumn v. spring	1.30624	0.44224	2.954	0.003140 **
Autumn v. summer	0.81817	0.37229	2.198	0.027974 *
*A. flavicollis* v. *Cl. glareolus*	0.28740	0.34491	0.833	0.404698
2019 v. 2020	0.23872	0.35038	0.681	0.495677
2019 v. 2021	−0.78207	0.43313	−1.806	0.070976
2019 v. 2022	−2.01711	1.04650	−1.927	0.053920 .
Adult v. juvenile	−1.31092	0.44867	−2.922	0.003480 **
Female v. male	0.09002	0.31743	0.284	0.776736
*Apodemus flavicollis*				
Intercept	−3.9593	1.1871	−3.335	0.000852 ***
Autumn v. spring	1.4047	0.9242	1.520	0.128515
Autumn v. summer	1.0298	0.7217	1.427	0.153607
Haselmuehl v. Heselbach	2.7111	1.0687	2.537	0.011184 *
2019 v. 2020	−0.5673	0.7925	−0.716	0.474066
2019 v. 2021	−1.8405	1.1040	−1.667	0.095504
2019 v. 2022	−1.7362	1.1382	−1.525	0.127161
Adult v. juvenile	−2.3298	1.1194	−2.081	0.037411 *
Female v. male	−1.2373	0.5951	−2.079	0.037597 *
*Clethrionomys glareolus*				
Intercept	−1.935855	0.330835	−5.851	4.87 × 10^−9^ ***
Autumn v. spring	0.524291	0.361727	1.449	0.1472
Autumn v. summer	0.494920	0.288599	1.715	0.0864
Haselmuehl v. Heselbach	−0.084538	0.266944	−0.317	0.7515
2019 v. 2020	0.001682	0.310668	0.005	0.9957
2019 v. 2021	−0.268220	0.399430	−0.672	0.5019
2019 v. 2022	−0.796784	0.540969	−1.473	0.1408
Female v. male	1.363863	0.289338	4.714	2.43 × 10^−6^ ***

Std. Error = Standard Error; v. = versus; Significance codes: 0 ‘***’ = extremely significant; 0.001 ‘**’ = highly significant; 0.01 ‘*’ = very significant; 0.05 ‘.’ = significant.

**Table 3:** The number of tick-borne encephalitis virus seropositive rodents of total sampled by years, seasons, and locations. Serum and thoracic lavage samples are considered together.

		TBEV Seropositive/Sampled Rodents																	
		Haselmuehl									Heselbach								
		*Clethrionomys glareolus*			*Apodemus flavicollis*						*Clethrionomys glareolus*			*Apodemus flavicollis*					
Year	Season	M	F	Juv.	M	F	Juv.	Total	Seropos. (%)		M	F	Juv.	M	F	Juv.	Total	Seropos. (%)	
									Y	S								Y	S
2019	Spring	3/5	2/19	2/6	0/8	0/2	0/0	7/40	16.8	17.5	2/7	5/16	0/3	1/2	3/5	0/3	11/36	19.8	30.6
	Summer	9/15	2/17	3/27	0/7	0/4	0/0	14/70		20.0	3/5	0/11	2/16	3/18	4/8	1/7	13/65		20.0
	Autumn	3/13	1/14	5/25	0/8	0/8	0/1	9/69		13.0	1/6	3/12	2/24	2/10	0/6	0/3	8/61		13.1
2020	Spring	0/0	0/0	0/0	0/0	0/0	0/0	0/0	9.1	0	1/4	0/0	2/3	0/0	0/0	0/0	3/7	23.4	42.9
	Summer	2/3	0/15	0/1	0/1	0/4	0/0	2/24		8.3	8/13	6/19	0/12	0/3	3/7	0/1	17/55		30.9
	Autumn	1/3	0/8	1/7	0/1	0/1	0/0	2/20		10.0	3/8	1/8	1/25	0/3	0/1	0/0	5/45		11.1
2021	Spring	0/0	0/0	0/2	0/0	0/0	0/0	0/2	11.4	0	0/5	4/6	0/0	0/0	0/0	0/0	4/11	12.0	36.4
	Summer	1/1	0/1	0/0	0/0	0/1	0/0	1/3		33.3	2/10	1/6	0/6	0/6	0/4	0/1	3/33		9.1
	Autumn	1/1	1/4	0/8	0/5	1/3	0/9	3/30		10.0	2/3	0/3	0/9	0/8	0/4	0/4	2/31		6.5
2022	Spring	0/4	0/2	0/0	0/0	0/0	0/0	0/6	31.3	0	0/2	0/1	0/0	0/0	0/1	0/0	0/4	3.0	0
	Summer	4/4	1/5	0/0	0/0	0/1	0/0	5/10		50.0	0/1	0/2	0/3	0/4	1/5	0/2	1/17		5.9
	Autumn	0/0	0/0	0/0	0/0	0/0	0/0	0/0		0	0/1	0/4	0/6	0/1	0/0	0/0	0/12		0
Total		24/49	7/85	11/76	0/30	1/24	0/10	43/274			22/65	20/88	7/107	6/55	11/41	1/21	67/377		
Seropos. (%)		49.0	8.2	14.5	0	4.2	0	15.7			33.8	22.7	6.5	10.9	26.8	4.8	17.8		

Y = Year; S = Season; M = Male; F = Female; Juv. = Juvenile; Seropos. = Seropositivity.

**Table 4:** Listing of seropositive rodents which were caught at least two times with the results of the indirect immunofluorescence assay (IIFA).

ID	Species	Sex	First Capture		Second Capture			Third Capture		
			Sample	IIFA	Days upon 2nd Capture	Sample	IIFA	Days upon 3rd Capture	Sample	IIFA
Seroconversion										
35_Heb	*Cl glareolus.*	f	serum	neg.	42	serum	pos.			
26_Heb	*Cl. glareolus*	f	serum	neg.	43	lavage	pos.			
31_Heb	*Cl. glareolus*	f	serum	neg.	42	lavage	pos.			
220_Heb	*Cl. glareolus*	f	serum	neg.	293	serum	pos.			
169_Heb	*A. flavicollis*	m	serum	neg.	59	serum	pos.			
250_Heb	*A. flavicollis*	f	serum	neg.	308	lavage	pos.			
224_Heb	*Cl. glareolus*	f	serum	neg.	47	serum	neg.	142	serum	pos.
194_Heb	*Cl. glareolus*	m	n.a.	n.a.	23	serum	neg.	293	serum	pos.
222_Heb	*Cl. glareolus*	m	serum	neg.	47	serum	neg.	246	serum	pos.
59_Ham	*Cl. glareolus*	m	serum	neg.	48	serum	pos.	36	serum	pos.
297_Heb	*Cl. glareolus*	f	serum	neg.	294	serum	pos.	37	serum	pos.
275_Heb	*A. flavicollis*	f	serum	neg.	244	serum	pos.	20	serum	pos.
465_Ham	*Cl. glareolus*	m	serum	neg.	62	serum	pos.	29	serum	pos.
Positive recaptured once										
20_Ham	*Cl. glareolus*	m	serum	pos.	92	serum	pos.			
21_Ham	*Cl. glareolus*	m	serum	pos.	92	serum	pos.			
146_Ham	*Cl. glareolus*	m	serum	pos.	59	serum	pos.			
84_Ham	*Cl. glareolus*	m	serum	pos.	92	serum	pos.			
105_Ham	*Cl. glareolus*	f	serum	pos.	59	serum	pos.			
232_Heb	*Cl. glareolus*	f	serum	pos.	48	serum	pos.			
388_Heb	*Cl. glareolus*	m	serum	pos.	29	serum	pos.			
426_Heb	*Cl. glareolus*	m	serum	pos.	30	serum	pos.			
290_Heb	*Cl. glareolus*	f	serum	pos.	17	serum	pos.			
261_Heb	*Cl. glareolus*	m	serum	pos.	141	serum	pos.			
283_Heb	*Cl. glareolus*	f	serum	pos.	17	lavage	pos.			
Positive recaptured twice										
16_Ham	*Cl. glareolus*	f	serum	pos.	42	n.a.	n.a.	50	serum	pos.
9_Ham	*Cl. glareolus*	m	serum	pos.	91	serum	pos.	36	serum	pos.
80_Heb	*Cl. glareolus*	m	serum	pos.	33	serum	pos.	59	serum	pos.
232_Heb	*Cl. glareolus*	f	serum	pos.	47	serum	pos.	142	serum	pos.
294_Heb	*Cl. glareolus*	m	serum	pos.	15	serum	pos.	21	serum	pos.
305_Heb	*Cl. glareolus*	m	serum	pos.	20	serum	pos.	36	serum	pos.
385_Heb	*Cl. glareolus*	f	serum	pos.	36	serum	pos.	28	serum	pos.
77_Heb	*A. flavicollis*	f	serum	pos.	33	serum	pos.	36	serum	pos.
Seropositive to seronegative captures										
189_Ham	*Cl. glareolus*	f	n.a.	n.a.	23	serum	pos.	48	serum	neg.
136_Heb	*A. flavicollis*	m	serum	pos.	36	lavage	neg.			
69_Heb	*A. flavicollis*	m	serum	pos.	16	n.a.	n.a.	91	serum	neg.

n.a. = not available (due to weakened physical conditions or the body weight was under 14 g); pos. = positive; neg. = negative.

## Data Availability

Not applicable.
